# Phenolic Profile, Antioxidant Capacities and Enzymatic Inhibitory Activities of Propolis from Different Geographical Areas: Needs for Analytical Harmonization

**DOI:** 10.3390/antiox9010075

**Published:** 2020-01-15

**Authors:** Sandra M. Osés, Patricia Marcos, Patricia Azofra, Ana de Pablo, Miguel Ángel Fernández-Muíño, M. Teresa Sancho

**Affiliations:** Department of Biotechnology and Food Science, Universidad de Burgos (University of Burgos), Pza. Misael Bañuelos s/n, 09001 Burgos, Spain; smoses@ubu.es (S.M.O.); pmg0035@alu.ubu.es (P.M.); pat0014@alu.ubu.es (P.A.); anadpablotorres@gmail.com (A.d.P.)

**Keywords:** propolis, phenolic profile, antioxidant capacities, hyaluronidase inhibitory activity, ACE inhibitory activity

## Abstract

Propolis is a resinous vegetal exudate modified by bees, and is interesting as a preservative and potentially functional product. This work dealt with studying the common phenolic profiles and antioxidant capacities of 13 bee propolis from different geographical areas. Both hyaluronidase and angiotensin converting enzyme (ACE) inhibitory activities were also assessed and related when possible with particular phenolic compounds. High performance liquid chromatography-ultraviolet detection (HPLC-UV) analysis showed that every propolis contained *p*-coumaric acid (1.2–12.2 mg/g) and ferulic acid (0.3–11.0 mg/g). Pinocembrin, catechin, and caffeic acid phenethyl ester (CAPE) plus galangin were the main flavonoids. Antioxidant activities were higher than 280 µmol trolox/g for trolox equivalent antioxidant capacity (TEAC), 0.099 mmol uric acid/g for radical-scavenging effect on hydroxyl radicals, and 0.19 mg/mL for half maximal inhibitory concentration (IC_50_) of antioxidant activity against superoxide anion radical. Working with solutions of 10 mg/mL propolis, hyaluronidase inhibitory activity ranged between 0% and 68.20%, being correlated to ferulic acid content. ACE inhibitory effect determined by HPLC was higher than 78%, being correlated with catechin and *p*-coumaric acid. Therefore, propolis could be useful for food, pharmaceutical, and cosmetic companies, also helping to reduce risk factors for diseases related to oxidative damage, inflammatory processes, and hypertension. This research also highlights the necessity for harmonized analysis methods and the expression of results for propolis.

## 1. Introduction

Propolis is a resinous product made by bees mixing plant exudates with beeswax and other substances, such as salivary secretions. Its composition varies depending on the geographic location, botanical origin, and climatic factors [1]. More than 300 compounds have been found in different propolis. Propolis is mainly composed of 40–70% balsam (flavonoids and phenolic acids), 1–3% essential oils, 20–35% waxes and 5% other substances (most of them coming from pollen or being provided by the bees), such as minerals, polysaccharides, and proteins [2]. To make propolis, bees use raw materials from different botanical species, depending on the flora available in each geographical area, which determines propolis chemical diversity. The basic plant source of European propolis (temperate zone), are the bud exudates of trees of the genus *Populus* sp., mainly black poplar *Populus nigra* L. [1], whose main constituents are flavonoids with an unsubstituted B-ring, esters of glycerol and cinnamic acids, being the sesquiterpenes the predominant substances within the volatile oil fraction [3]. The main botanical source of Brazilian propolis (tropical zone), is the Asteracean shrub *Baccharis dracunculifolia* DC. [4], in which terpenoids and prenylated derivates of *p*-coumaric acids prevail [5].

Propolis has been used since ancient times as an antiseptic and cicatrizing agent for wounds due to its properties. The potential bioactive properties of propolis, such as antioxidant, antimicrobial, anti-inflammatory, antiulcer, wound healing, anti-angiogenin, and anti-hyperlipidemic, have been attributed to phenolic compounds, among which flavones, flavonols, flavanones, dihydroflavonols, caffeic acid phenethyl ester (CAPE), and cinnamic acids and their esters are included [6,7,8,9]. Nowadays, propolis is used for the growth performance improvement of livestock, as well as for several purposes in food industry, biomedical applications, oral hygiene and cosmetic products [10]. Because of its promising functional properties, there is currently a growing interest in commercializing propolis.

Until June 2019, fifteen health claims related to bee propolis were received by the European Commission [11] alleging the following effects: “antioxidant properties”, “respiratory health”, “antibacterial and antifungal activities”, “throat comfort”, “gut health”, “supports immune defenses”, “maintenance of oral health”, “helps to maintain a normal blood circulation” and “hepatoprotective”. All health claims were rejected because of probable qualitative and quantitative variations of flavonoids depending on raw materials (different botanical and geographical sources), and the extraction and preparation methods. The panel of assessors also considered that no evidence proved a beneficial physiological effect. All the above occurred because research has been mainly focused on highlighting the differences among propolis of different origins and different bees, so far. Nevertheless, taking a new approach to establish similarities of propolis from different countries is of paramount importance and could help to overcome current limitations, with a positive economic impact on the beekeeping sector.

So far, published research on propolis lacks the necessary harmonization regarding common extraction procedures, common reference standards and, what is more important, common results’ expression, making it impossible data comparison among studies. Moreover, literature on antioxidant activity of propolis is mainly focused on one or two general procedures, such as ferric reducting antioxidant power (FRAP), 2,2-diphenyl-picrylhydrazyl assay (DPPH) and/or trolox equivalent antioxidant capacity (TEAC), whose results are correlated [12]. Published research on propolis seldom evaluates antioxidant activity against specific free radicals of physiological interest. Thereby, published data about propolis antioxidant capacity are insufficient to estimate a broad spectrum of the actual propolis antioxidant activity.

The aims of this study were: first, to research possible common phenolics’ profiles of bee-propolis from distant geographical areas; second, to assess for first time, a broad spectrum of antioxidant capacity by determining TEAC (the most reliable procedure adaptable to measure both lipophilic and hydrophilic antioxidants based on a single electron transfer reaction mechanism [12]), and antioxidant activities against hydroxyl and superoxide radicals that are important from a physiological point of view, and third, to evaluate hyaluronidase and angiotensin converting enzyme (ACE) inhibitory activities, eventually studying possible correlations between each biological capacity and a particular phenolic group or phenolic compound.

## 2. Materials and Methods

Gallic acid and catechin from Panreac (Barcelona, Spain). CAPE and galangin from TargetMol (Boston, EEUU). Apigenin, chlorogenic acid, kaempferol and pinocembrin from Cymit Quimica, S.L. (Barcelona, Spain). The other standards are from Sigma–Aldrich (Stein-heim, Germany).

This study was carried out on 13 propolis samples harvested in 2015 that came from different geographical areas (6 samples from North-East European countries [P1–P6], 2 samples from the South American tropical zone [P7–P8], and 5 samples from South-West European countries [P9–P13]). All samples were stored in the dark at −20 °C until use.

Propolis extraction was performed accordingly to the harmonized procedure established within the propolis working group of the International Honey Commission: propolis samples were grounded in a marble mortar at −30 °C. One gram of pulverized propolis sample was weighted and 30 mL of 70% food grade ethanol were added. The mixture was kept under mechanical agitation at room temperature and in the absence of light for 24 h. Then, the mixture was filtered (Whatman filter paper No. 4), and the solid was re-extracted in the same conditions as reported. Extraction procedure was repeated until getting absence of phenolics (no colour development) in the solids, adding a few drops of FeCl_3_ (5% in methanol). After the third or fourth extraction, all the extracts were combined in a 100-mL volumetric flask and the volume was adjusted with 70% ethanol/water. The extraction procedure was performed in triplicate for each sample and the total volume for each sample (300 mL), was mixed in an amber glass bottle and kept frozen until further use.

Total phenolics were determined in the ethanolic extracts by the method based on the reaction of phenolics with Folin-Ciocalteu reagent [13]. Absorbance was measured at 760 nm, using gallic acid as a standard for the calibration curve. Results were expressed as mg of gallic acid (GA)/g propolis.

Flavone/Flavonol content was analysed by the reaction of these flavonoids with AlCl_3_ in neutral medium [13]. Absorbance was read at 425 nm, using quercetin as standard for the calibration curve. Results were expressed as mg of quercetin (Q)/g of propolis.

Flavanones/dihydroflavonols content was determined by the reaction of these flavonoids with dinitrophenol [14]. Absorbance was measured at 486 nm, using naringenin as standard for the calibration curve. Results were expressed as mg of naringenin (N)/g of propolis.

For the determination of flavan-3-ols (catechin, rutin, luteolin), these compounds reacted with AlCl_3_ in alkaline medium [15]. Absorbance was read at 510 nm, using catechin as standard for the calibration curve. Results were expressed as mg of catechin (C)/g of propolis.

**Figure 1:**
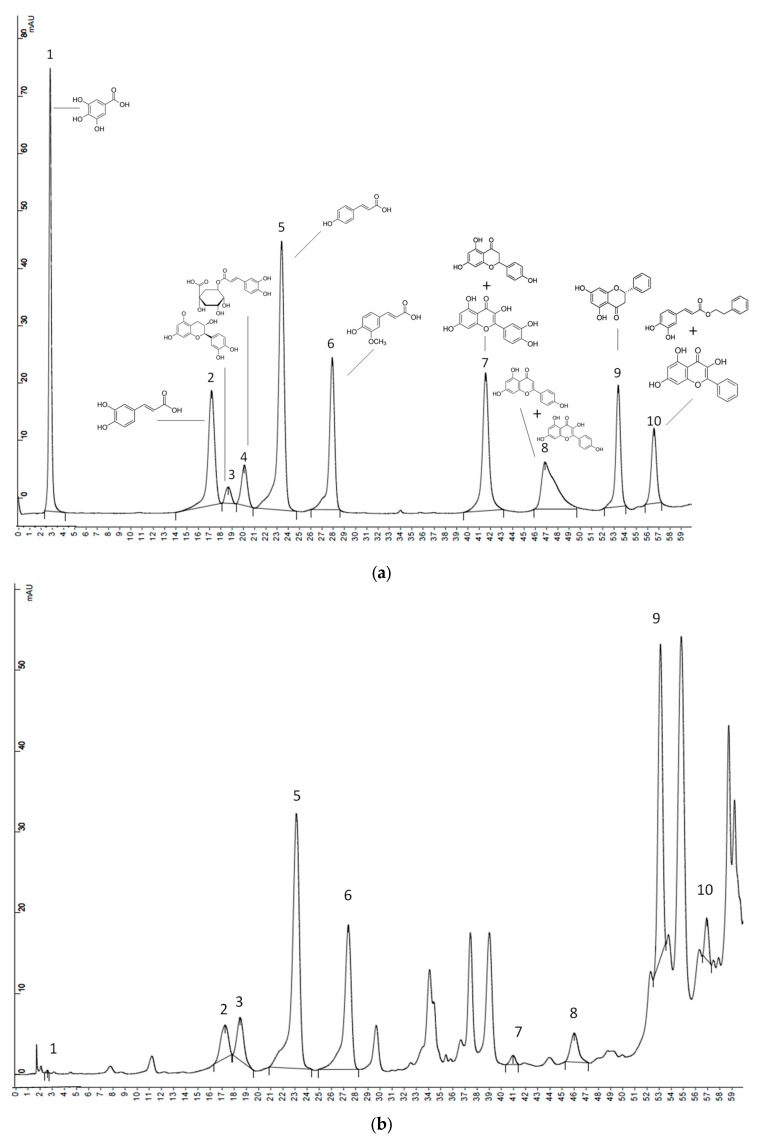
Chromatograms of the standard phenolic compounds (**a**) and NE-Europe propolis sample (**b**) obtained by high performance liquid chromatography-ultraviolet detection (HPLC-UV). Gallic acid (1), caffeic acid (2), catechin (3), chlorogenic acid (4), *p*-coumaric acid (5), ferulic acid (6), naringenin + quercetin (7), apigenin + kaempferol (8), pinocembrin (9), caffeic acid phenethyl ester (CAPE) + galangin (10).

Ethanol extracts of the propolis samples were purified through polyvinylidene difluoride (PVDF) 0.45 µm filters (Whatman^TM^ GE Healthcare, Buckinghamshire, UK) and analyzed by high performance liquid chromatography-ultraviolet detection (HPLC-UV) [16,17], using a chromatograph Varian Pro Star 310 (Varian, Victoria, Australia). 20 µL sample was injected. The chromatographic separation was carried out on a reversed-phase Microsorb-MV 100-5 C18 column (150 × 4.6 mm, 5 µm particle size) provided by Agilent Technologies (Agilent, Santa Clara, CA, USA). The mobile phase comprised (A) 0.1% formic acid in miliQ water and (B) 0.1% formic acid in acetonitrile. The solvent gradient was: 0–7 min, 0% B, 7–12 min, 2% B, 12–20 min, 8% B, 20–23 min, 10% B, 23–33 min, 20% B, 33–45 min, 23% B, 45–50 min, 30% B, 50–55 min, 32% B, and 55–60 min, 50% B. The flow rate was 1 mL/min and UV detection was carried out at 280 nm. Quantification was carried out using calibration curves at eight concentration levels (0.0005–0.5 mg/mL). The linearity of all compounds was satisfactory with *R*^2^ values > 0.9925. Limits of detection ranged from 0.0001 mg/mL to 0.0049 mg/mL. Limits of quantification varied between 0.0009 mg/mL and 0.0163 mg/mL (Supplementary Table S1). There were compounds that eluted at the same detection time. Therefore, they were quantified together: naringenin + quercetin, apigenin + kaempferol and CAPE + galangin (Figure 1).

Trolox equivalent antioxidant capacity (TEAC) of ethanolic propolis extracts was evaluated mixing 1490 μL of ABTS^•+^ (generated mixing 1:1 ABTS with K_2_S_2_O_8_ and keeping at dark during 16–18 h) with 10 μL of sample, standard or blank. Trolox was used as standard for the calibration curve (0.625–3 mM). The percentage inhibition after 6 min was calculated [18]. Results were expressed as μmol Trolox (T)/g of propolis.

Antioxidant capacity as radical-scavenging effect on hydroxyl radicals (AOA assay) was measured by quantifying the ability of propolis extracts to inhibit the synthesis of thiobarbituric acid and reactive substances (TBARS) from sodium benzoate under the influence of free radicals produced by Fenton’s reaction [17,19]. Ethanolic propolis extracts (10 μL) were mixed with 490 μL of sodium phosphate buffer (0.1 M, pH 7.4), 500 μL of sodium benzoate (0.01 M), 200 μL of FeSO_4_-EDTA (2 mM) and 200 μL of hydrogen peroxide (0.01 M). After 1 h incubation at 37 °C, the reaction was stopped adding 1 mL of acetic acid (20%), adding later 1 mL of thiobarbituric acid (0.8% *w*/*v*) in NaOH (50 mM). The solution was boiled throughout 10 min and then cooled in ice. The absorbance was measured at 532 nm against distilled water. Each sample (A1) had its own control (A0), in which acetic acid (20%) was added before Fe-EDTA and H_2_O_2_. For each series of analysis, a negative control (K1 and K0) was prepared, where the samples had been replaced with phosphate buffer. Further, 1 mM uric acid in NaOH (5mM) (U1 and U0) was used as standard. Antioxidant activity was calculated as mmol uric acid (UA)/g of propolis = 0.1 × (CU) × (K − A)/(K − U), where CU is the concentration of uric acid (1 mM), K is the absorbance of control (K1-K0), A is the absorbance of sample (A1-A0) and U is the absorbance of uric acid solution (U1-U0).

Superoxide anion radical was generated by the xanthine-xanthine oxidase system [20]. In an Eppendorf tube (Eppendorf Ibérica, Madrid, Spain), 0.48 mL of 0.05 M sodium carbonate buffer (pH 10.5), 0.02 mL of 3 mM xanthine, 0.02 mL of 3 Mm EDTA, 0.02 mL of 0.15% bovine serum albumin (BSA), 0.02 mL of 0.75 mM nitroblue tetrazolium (NBT) and 0.02 mL of sample were mixed. After 10 min at 25 °C xanthine oxidase 6 mU was added to start the enzymatic reaction that was carried out at 25 °C during 20 min. Then, the reaction was stopped by adding 0.02 mL of 6 mM CuCl. The absorbance was measured at 560 nm against a blank for each sample where the enzyme was not added. Half maximal inhibitory concentration (IC_50_) values for the inhibition of the generation of superoxide anions by the propolis extracts were calculated by measuring the reduction of NBT to form blue formazan.

Hyaluronidase inhibitory activity was assessed by hyaluronidase inhibition assay [21] based on the mechanism of the Morgan-Elson reaction with few modifications. A stock solution of 5 mg/mL hyaluronic acid sodium salt from *Streptococus equi* was prepared in water and stored at 4 °C. Hyaluronic acid stock solution (70 μL) and 100 μL of buffer (0.2 M sodium formate, 0.1 M NaCl and 0.2 mg/mL BSA, pH adjusted to 3.68 with formic acid) were added to 200 μL milliQ water and 50 μL sample. The mixture was heated at 37 °C for 10 min before starting the reaction by addition of 50 μL of hyaluronidase from bovine testes type IV-S (1400 U/mL) prepared in 0.9% NaCl. The mixture was incubated for 1 h at 37 °C in a water bath. The enzymatic reaction was stopped by adding 100 μL of 0.8 M potassium tetraborate and then, incubated 3 min in water-bath at ebullition. The test tubes were cooled at room temperature and 750 μL of *p*-dimethylaminobenzaldehyde (DMAB) was added (DMAB was prepared dissolving 2 g of DMAB in a mixture of 2.5 mL of 10 N HCl and 17.5 mL of glacial acetic acid, further diluted 1:2 with glacial acetic acid immediately before use). The tubes were incubated for 20 min at 37 °C and the colour of the resulting product was read at 586 nm against a blank sample (where enzyme and sample had been substituted by buffer). Enzyme activity was defined as 1 unit (U) of hyaluronidase that catalyzes the liberation of 1 μmol *N*-acetyl-d-glucosamine (NAG) per min under specified conditions. NAG standard solutions (in the range between 0 and 2 μmol per test), were used as standard for calibration curves. With the NAG formed in each enzymatic reaction and using the linear regression equation, the percentage of enzyme inhibition was calculated as % Inhibition = (A − B)/A × 100, where A was μmol of NAG in the positive control (where μL of sample was substituted by buffer) and B was μmol of NAG of each sample reaction.

ACE inhibitory activity was determined by HPLC-UV [22]. The method was based on the hydrolysis of *N*-hippuryl-histidyl-leucine (HHL) into hippuric acid (HA) and His-Leu (HL) by the angiotensin converting enzyme (ACE). Propolis sample (10 μL) was mixed with 30 μL of ACE (60 mU/mL) prepared in buffer (sodium chloride 0.3 M, potassium phosphate 0.1 M pH 8.3) and incubated 10 min at 37 °C. Then, 90 μL of HHL solution (5 mM), pre-incubated at 37 °C, was added to tubes and the mixture was incubated 1 h at 37 °C. To stop the reaction, 8 μL of 5 M HCl was used. After enzymatic reaction, each sample was filtered through 0.2 μm PVDF filter unit (WhatmanTM GE Healthcare, Buckinghamshire, UK). The concentration of HA released in the enzymatic reaction was determined on a HPLC system (Pro Star Varian, Victoria, Australia), using a C_18_ Ultrasphere ODS column (4.6 mm × 250 mm) (Beckmann^®^, Indianapolis, USA). 25 μL sample was injected. Mobile phase was a solution of acetonitrile 12.5% and trifluoroacetic acid 0.1% in water. Flow rate was 1 mL/min and a Pro star 325 UV-Vis detector measuring the optical density at 228 nm during 15 min. Data were analysed using Star Chromatography Workstation version 6.41 software (Varian, Victoria, Australia). External standard solutions of HA (1–1000 μM) were used for the calibration curve. Each day of analysis, a blank (without enzyme and sample), and a control (without sample), were also injected. Furthermore, each sample had its own blank, where the enzyme was substituted by buffer. The results were expressed as % inhibitory ACE activity = ([HAcontrol] − [HAsample])/[HAcontrol] × 100%.

Each assay was carried out in triplicate. All results were evaluated by multiple range tests assessed by Turkey HSD test (*p* < 0.05). Pearson correlations were applied to the results. Statistical software Stagraphics Ceturion XVII (Statgraphics Technologies, Inc., The Plains, VA, USA)) was used.

## 3. Results and Discussion

### 3.1. Total Phenolic Content

The phenolic content of propolis ranged between 65.49 mg GA/g and 228.40 mg GA/g. Literature references describe a variety of ranges for total phenolics of propolis from different geographical origins, depending on both standard and solvent used. Operating with gallic acid as standard and methanol as solvent/diluent, total phenolics of Portuguese and Brasilian propolis ranged from 29.5 to 137 mg/g [23,24]. Using gallic acid as standard and ethanol as solvent, higher amounts of phenols were obtained (150–340 mg/g), for propolis from Spain [25,26], Poland [27] and China [28]. Employing pinocembrin as standard, a phenolic content of 62.7 mg/g was found for Portuguese propolis [29]. Utilizing chlorogenic acid as standard, amounts of 287 mg/g were obtained in Slovenian propolis [30]. Using caffeic acid as standard, total phenolics’ values ranged from 0.74 to 338 mg/g in propolis from Morocco [31] and Greece [32]. A total phenolic content between 30 and 329 mg/g was obtained when a mixture of caffeic acid:galangin:pinocembrim (1:1:1) was used as standard for Portuguese [33] and Brasilian [34] propolis. Our results also showed a great variability. However, it is important to highlight the fact that, regarding propolis’ total phenolic content, the comparison of data is very difficult because, on the one hand, there is not a consensus standard for the calibration curve (gallic acid, caffeic acid, chlorogenic acid, pinocembrin, galangin, and mixes of them are used in different studies), and on the other hand, there is still a worrisome lack of regulation regarding standardized procedures, although reliable standard methods for *Apis mellifera* propolis were proposed [35].

### 3.2. Total Flavonoids Content

In respect of flavones and flavonols, our values ranged between 18.48 mg Q/g and 83.76 mg Q/g. Flavanones and dihydroflavonols ranged between 25.07 and 128.46 mg N/g. As for flavanols, values ranged between 27.89 and 108.18 mg C/g. Our results of flavone and flavonols as well as flavanones and dihydroflavonols were similar to those obtained by other researchers in propolis from a variety of geographical origins, with results of 13–62 mg Q/g flavone and flavonols, and 55–100 mg N/g flavanones and dihydroflavonols [23,26,27,28,29,36]. Flavanones were described as the main constituents of poplar-type propolis [14]. However, in this study the results for all flavonoids’ groups were quite similar. Flavanols were hardly researched by spectrophotometric assays. Similar values than ours were obtained for Ethiopian propolis, ranging from 14.76 to 68.88 mg C/g [37] and lower for Thai propolis, with an average of 3.40 mg C/g [38].

### 3.3. Quantification of Phenols Compounds Using HPLC-UV

Figure 1 shows two chromatograms: (a) corresponds to a mixture of phenolics’ standards and (b) to the phenolic profile of one sample from NE-Europe. Table 1 shows the phenolic compounds (mg/g), quantified in propolis by HPLC-UV. In our study, gallic acid was only quantified in three samples and chlorogenic acid in six samples. Flavonoids were found in higher concentration than phenolic acids, being catechin and pinocembrin the main flavonoids. Our North-East European propolis showed all phenolic compounds, except gallic and chlorogenic acids, being catechin and pinocembrin together with CAPE + galangin the major compounds. In literature references, Slovenian propolis showed the lowest results for gallic acid, and trace concentrations of chlorogenic acid [30]; Hungarian propolis had pinocembrin and chrysin as the major phenolics, being chlorogenic acid also described [39]. Polish propolis [40] were characterized by *p*-coumaric acid, 2-acetyl-1,3-di-*p*-coumarylglycerol, and *p*-coumaric acid benzyl ester together with galangin and chrysin as the main polyphenols. South-West European propolis didn’t contain gallic acid, but had higher amounts of pinocembrin and caffeic acid, and lower content of ferulic acid than our other propolis. In Italian and Spanish propolis, different studies showed that pinocembrin was one of the most important flavonoids and isoferulic, ferulic and caffeic acids were the major phenolic acids [41,42,43]. Two samples of propolis from South America showed different phenolic compositions, probably because they were from different botanical and geographical origins or were harvested in different seasons [44], being *p*-coumaric acid the major phenolic compound in one of the samples, and catechin, CAPE and galangin the major phenolics in the other sample.

Even although propolis samples exhibited different phenolics’ profiles agreeing with literature [40], two acids: *p*-coumaric (1.2–12.2 mg/g propolis) and ferulic (0.3–11.0 mg/g propolis) were found in all of our samples, regardless their origin. Therefore, research about biological activities related to these two acids is of great interest in order to provide with some of the necessary evidences for future proposals regarding propolis’ health claims. All propolis also exhibited quantifiable values for the sums of naringenin + quercetin (0.3–3.2 mg/g propolis), apigenin + Kaempferol (0.6–32.2 mg/g propolis) and CAPE + galangin (2.4–110.6 mg/g propolis), but in this case, given that the sum of two compounds was involved, it was not possible to define if one or both compounds were common to all samples. Apart from the thirteen standard compounds quantified (Figure 1b), some other non-identified peaks were present in the samples, which is usual, due to the huge variety of phenols described in the literature in propolis around the word [30,39,40,41,42,43,44].

With regard to the similarities or differences of our results for individual compounds with those of the literature references, it is essential to point out that the comparison is extremely difficult because the concentration units are different. One research gave the results as percentage of peaks’ areas [40], while other studies showed the values as µg or mg compound/g propolis extract or balsam [30,39,41,43,44], and another one expressed the results as mg/g, not specifying if the denominator referred to g extract (or balsam) or to g propolis [42], although it is likely that it was g propolis, because in the units for other phenolic-related parameters of the same study the denominator referred to g propolis [42]. The lack of uniformity regarding analytical procedures and expression of results highlights the urgency of the development of a legal regulation for propolis.

### 3.4. Antioxidant Capacities

**Figure 2:**
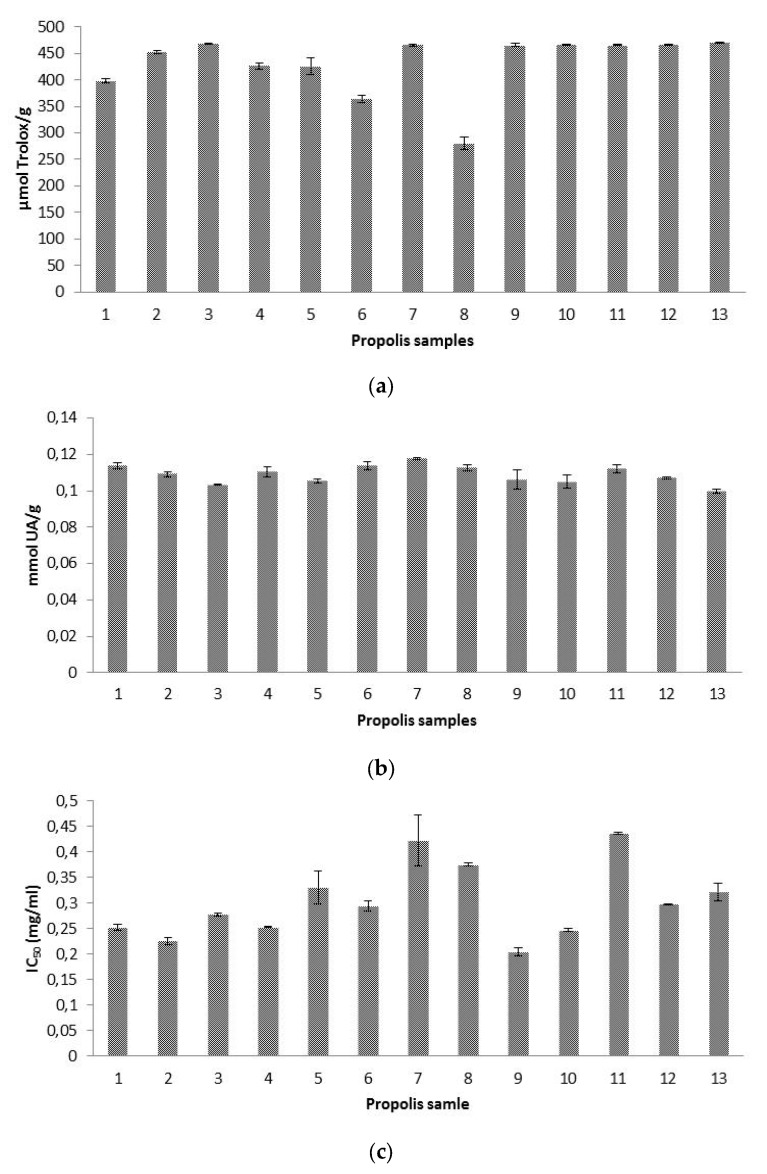
Mean values (*n* = 3) of: (**a**) Trolox equivalent antioxidant capacity (TEAC) expressed as μmol Trolox/g of propolis, (**b**) radical-scavenging activity on hydroxyl radicals expressed as mmol uric acid (UA)/g of propolis, and (**c**) radical-scavenging effect on superoxide radicals expressed as half maximal inhibitory concentration (IC_50_) (mg/mL). Error bars represent the standard deviation for each data point.

Antioxidant capacities of our propolis ethanolic extracts were assessed for the first time against three different free radicals, namely ABTS^•+^, hydroxyl radical, and superoxide anion radical. This important from the point of view of assessing a broad spectrum of antioxidant activity of this product as explained in the introduction section. The results are shown in Figure 2.

#### 3.4.1. Antioxidant Capacity by ABTS^•+^ Scavenging Activity Test

Results of trolox equivalent antioxidant capacity (TEAC) ranged between 280 and 470 µmol T/g propolis. Like the above-mentioned case of the contrasting units found in literature references for the concentration of individual phenolic compounds, the same problem occurs with regard to literature TEAC values, aggravated by the use of different reagents and alcohol concentrations. Higher TEAC results than ours were obtained in Spanish propolis [25,26], but the results were shown as µmol T/g extract. Moreover, in one of the references [25], 90% ethanol was used for balsam extraction, in contrast to the 70% ethanol used for our present research. It was claimed that the higher the concentration of ethanol was, the higher the antiradical activity of propolis extracts was, being also this activity significantly affected by pH of the solvent used [27]. Using 80% ethanol as extracting agent, in Brazilian propolis, ranges between 25.5 and 439.2 µmol T/g sample were obtained, not specifying if the denominator referred to g extract or g propolis, but being likely to be g propolis, because in the same study and regarding DPPH antioxidant activity, the denominator referred to g propolis [45].

In order to check possible synergistic or antagonistic effects among single phenolic compounds detected in our propolis samples, antioxidant activity against ABTS^•+^ radical was assessed for individual commercial standards of phenolic compounds (Supplementary Table S2). For each sample, the TEAC result corresponding to the addition of the TEAC value provided by each assayed compound quantified by HPLC-UV was calculated (Supplementary Table S3). The TEAC value for each phenolic standard was different, agreeing with a previous study in which antioxidant activity of phenolic acids and flavonoids derived from propolis was claimed to be dependent on their structure, number of hydroxyl groups in their molecules, steric effects, position of hydroxyl groups, as well as the substituents on the aromatic ring, the double bond between C-2 and C-3 and the presence of the 4-oxo group, among other factors [46]. In general, our propolis samples showed antioxidant activities higher than those resulting from the addition of phenolic compounds quantified by HPLC-UV, so that the actual antioxidant activity of propolis appears to be linked to the synergistic effect of different phenolic compounds, and to the effect of other non-phenolic antioxidant substances. Ferulic acid is likely to be the main phenolic compound responsible for propolis TEAC, because it is present in all samples and exhibits a high TEAC value. It is probable that catechin also has an important contribution to TEAC, because of its relatively high concentration in the vast majority of propolis samples.

#### 3.4.2. Antioxidant Capacity as Radical-Scavenging Effect on Hydroxyl Radicals (AOA Assay)

Our propolis showed a scavenging ability against hydroxyl radical ranging between 0.099 and 0.117 mmol UA/g propolis (0.99–1.17 mM UA), which was slightly higher than that of the uric acid standard (1 mM). Values between 0.053 and 0.068 mmol UA/g propolis were obtained in Tunisian propolis [16]. Hydroxyl radical scavenging activity was also researched in other propolis using different units for the expression of results, what makes impossible a comparison among data, underlining the urgent need for an agreement regarding methodology and results’ expression. In two papers, hydroxyl radical scavenging activity was quantified as percentage inhibition rate with a 76.7% inhibition rate for Chinese propolis [47] and values ranging from 40–80% for Brazilian propolis [48].

#### 3.4.3. Antioxidant Capacity as Superoxide Radical-Scavenging Activity

Our antioxidant activity against superoxide anion radical showed IC_50_ values between 0.20 and 0.44 mg/mL. Higher superoxide scavenging activities were obtained in propolis from Algarve (southern Portugal) with IC_50_ values ranging from 0.01 to 0.053 mg/mL in methanolic extracts [36]. Using a propolis concentration of 75 mg/mL, our inhibition rates (77.41–100%), were similar to those obtained in Brazilian aqueous extracts, with values of 86.1% at 5 mg/mL, and 100% at 50 mg/mL [49], and in Japanese ethanolic extracts, with results around 80% at a concentration of 1% [20]. However, we consider that the comparison of results is not reliable enough, because extraction procedures and reagents used are different in each study.

With regard to the same antioxidant activity, no significant differences were found among our geographic groups (*p* > 0.05), which demonstrate that all propolis exhibited excellent antioxidant activities regardless their botanical and geographical origins.

### 3.5. Hyaluronidase Inhibitory Activity

**Figure 3:**
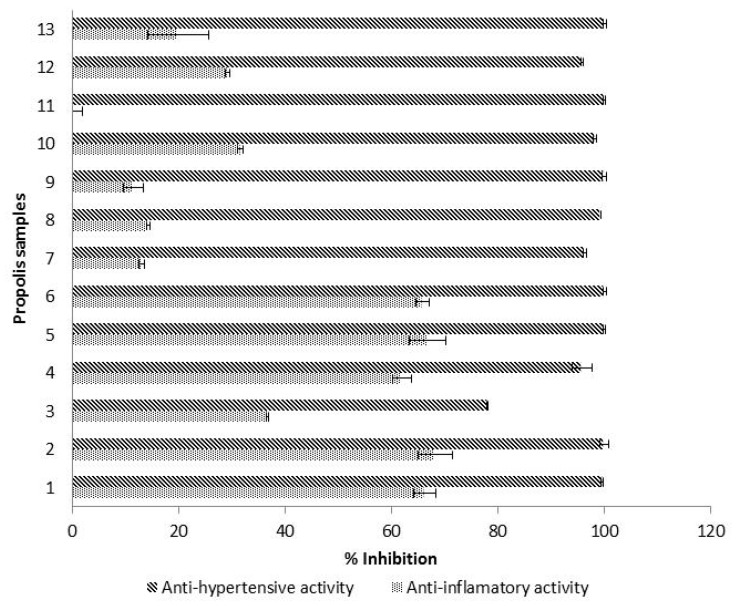
Mean values (*n* = 3) of hyaluronidase inhibitory activity and ACE inhibitory activity, expressed as % of inhibition. Error bars represent the standard deviation for each data point.

Hyaluronic acid is a component of articular cartilage that plays an important role in tissues’ renovation. Its degradation, by the hyaluronidase enzyme, is likely to cause pain, inflammation and bone loss [50]. Therefore, the quantification of hyaluronidase inhibition is an indirect way to assess the anti-inflammatory activity. In the studied propolis, hyaluronidase inhibitory activity ranged from 0% to 68.20% with a propolis concentration of 10 mg/mL (Figure 3). Similar results were observed by other authors. Inhibition rates between 10–20% were obtained for Portuguese propolis at 10 mg/mL, and between 53.76% and 75.79% at a concentration of 25 mg/mL [51]. Around 9% inhibition was reported for Brazilian propolis from stingless bees at 10 mg/mL and a 43.06% anti-inflammatory activity at 75 mg/mL [52]. The literature shows that anti-inflammatory activity values are highly dependent on the concentration of propolis working solutions such that, similar to other parameters previously mentioned (such as phenolics’ concentrations and antioxidant activities), it is of the utmost importance to standardize the analysis method for hyaluronidase inhibition in the next future.

In our study, the samples with low hyaluronidase inhibition values also showed low results for ferulic acid contents and for flavanones/dihydroflavonols concentrations. Therefore, it is likely that both ferulic acid and the group flavanones/dihydroflavonols are the main compounds responsible for a potential anti-inflammatory activity of propolis.

### 3.6. ACE Inhibitory Activity

The ACE inhibitory activity of propolis samples (10 mg/mL), expressed as % of inhibition of angiotensin I converting enzyme (ACE) is shown in Figure 3. ACE inhibitory activity was higher than 95% for all the samples except for one propolis (78%), which contained the lowest amount of flavanols (27.89 mg C/g). Our results agreed with literature, because a previous study carried out on Tunisian propolis also showed an ACE inhibition higher than 90% [17]. In comparison with other products, propolis exhibited a considerably higher ACE-inhibitory activity percentage, because values obtained for fermented milk (20–90%) [22], honeys at 50% (17–71%) [53], *Echium vulgare* honeys at 50% (94.2%) [54], mung bean and rice protein hydrolysates (52–96%) [55] as well as Ramie (*Boehmeria nivea* Gaudich) leaves extracts (51% inhibition) [56], were considerably lower. Therefore, the use of propolis could modulate the ACE, providing an effective control of hypertension, which is an important strategy to decrease the risk for cardiovascular diseases.

In pharmacies, the main compounds with ACE inhibitory activity are captopril-related antihypertensive drugs. However, these synthetic ACE inhibitors are known to produce cough, skin rashes and angioedema [57]. For this reason, it is of paramount importance to research other substances, especially natural products that could inhibit ACE to some extent. Most studies are focussed in peptides obtained from different food proteins, such as egg white [58], soybean [59], seafood [60,61] and other protein rich foods. Other studies show that some phenolic compounds, such as flavonoids, also exhibit ACE inhibition activity [62], so flavonoid extracts or products rich in flavonoids might be used against hypertension. With regard to propolis, further studies are still necessary to research the ACE inhibitory action at various concentrations in order to check if this activity depends on propolis concentration, as it was described for other products [60,61]. It is also necessary to perform more in vivo studies, similar to the study investigating Brazilian green propolis for the prevention of hypertension in rats [63].

In this study, higher values of ACE inhibition were found in samples with higher amounts of catechin and *p*-coumaric acid, so these two phenolic compounds could be responsible for a possible antihypertensive capacity of propolis. Indeed, catechin together with other flavonoids have been described as effective ACE inhibitors [64].

### 3.7. Correlations

All propolis showed antioxidant, hyaluronidase inhibitory and ACE inhibitory activities that could be due to different compounds or to the synergy of different constituents. Different compounds were reported to display the same activity, sometimes in the same order of magnitude [1]. Total phenolics exhibited a positive correlation with total flavones/flavonols (*r* = 0.7193). TEAC was positively correlated with total phenolics (*r* = 0.7151) and total flavones/flavonols (*r* = 0.5282), agreeing with other researchers’ results for propolis from Portugal, Morocco and Spain [26,29,31], showing furthermore in our study a positive correlation with caffeic acid concentration (*r* = 0.5171). Negative correlations were obtained between the antioxidant activity against hydroxyl radical and total phenolics (*r* = −0.5563) and total flavones/flavonols (*r* = −0.7101). Thus, it is likely that antioxidant activity against hydroxyl radicals could be more related to other propolis compounds, such as vitamins, proteins, organic acids, and minerals [2]. Hyaluronidase inhibitory activity was correlated with ferulic acid (*r* = 0.7245). It is important to highlight the fact that ferulic acid was one of the phenolic compounds common to all of our samples, and even although this acid was not described as efficient on cell proliferation inhibition [65], it should be deeper researched as an interesting anti-inflammatory related compound. Hyaluronidase inhibitory activity was also moderately correlated with total flavanones/dihydroflavonols (*r* = 0.3040). Samples with different polyphenols’ concentrations showed an almost equal hyaluronidase inhibitory activity, suggesting that these compounds were not the unique factors responsible for this activity [51]. As for ACE inhibitory activity, in a previous study carried out on honeys no significant correlations were observed between ACE inhibitory activity and phenols or antioxidant capacity [53]. In our research on propolis, ACE inhibitory activity was negatively correlated with total flavones/flavonols (*r* = −0.5451) and total flavanones/dihydroflavonols (*r* = −0.5540) and positively correlated with flavanols (*r* = 0.3766), catechin (*r* = 0.6096) and *p*-coumaric acid (*r* = 0.5575). This last correlation is interesting since *p*-coumaric acid was another phenolic compound that was present in all the samples of our study. It is likely that flavanols in synergy with *p*-coumaric acid and other propolis components are responsible for a potential antihypertensive activity. It is also important to highlight that some other phenolic compounds, not identified in this study but present in the propolis samples, could be responsible for these activities.

## 4. Conclusions

This study highlights the importance of setting up and proposing harmonized propolis analysis methods and the expression of results for phenolic compounds and antioxidant capacities, since common extraction procedures, reagents, and the expression of results are essential for efficient data comparison. With respect to the expression of results, we propose referring all values to g propolis to overcome differences in the extraction procedure and final extract concentration among different methods.

HPLC-UV analysis showed that *p*-coumaric and ferulic acids were identified in all samples. All propolis also exhibited quantifiable values for the sums of naringenin+quercetin, apigenin+kaempferol and CAPE+galangin. Flavonoids were found in higher concentration than phenolic acids, with pinocembrin, catechin, caffeic acid phenethyl ester (CAPE), and galangin being the main flavonoids.

Regardless of their origins, all propolis exhibited strong TEAC, hydroxyl, and superoxide radicals, meaning that they could be used to prevent product spoilage in food, pharmaceutical, cosmetic, and other companies. They might also play an important physiological role because of their capacity to scavenge hydroxyl and superoxide radicals related to cell damage and related diseases.

Solutions of 10 mg/mL propolis showed both hyaluronidase and ACE inhibitory activities. Hyaluronidase inhibitory activity was positively correlated with ferulic acid and to less extent to flavanones/dihydroflavonols. ACE inhibitory activity was positively correlated with catechin and *p*-coumaric acid, and to less extent to flavanols. The potent effect of propolis to inhibit ACE underlines its interesting antihypertensive potential.

## Figures and Tables

**Table 1:** Phenolic compounds (mg/g propolis) quantified by HPLC-UV in propolis from different geographical origins.

Zone	Sample	Gallic Acid	Caffeic Acid	Catechin	Clorogenic Acid	*p*-Coumaric Acid	Ferulic Acid	Naringenin + Quercetin *	Apigenin + Kaempferol *	Pinocembrin	CAPE + Galangin *
RT (min)		2.81	17.23	18.71	20.17	23.47	27.98	41.64	46.93	53.45	56.63
NE-Europe	1	0.3 ± 0.0 ^a^	0.7 ± 0.1 ^a^	29.1 ± 0.5 ^e^	ND	9.5 ± 0.2 ^bc^	11.0 ± 0.2 ^e^	0.4 ± 0.0 ^a^	4.5 ± 0.1 ^ab^	4.6 ± 0.3 ^b^	14.8 ± 0.4 ^de^
NE-Europe	2	0.3 ± 0.0 ^a^	2.2 ± 0.1 ^b^	19.8 ± 1.1 ^d^	ND	8.1 ± 0.3 ^b^	7.9 ± 0.3 ^d^	0.5 ± 0.0 ^bc^	15.8 ± 0.9 ^d^	16.5 ± 0.3 ^d^	5.8 ± 0.4 ^ab^
NE-Europe	3	ND	6.4 ± 0.5 ^e^	0.9±0.2 ^a^	1.4 ± 0.7 ^a^	2.3 ± 0.6 ^a^	1.4 ± 0.1 ^abc^	0.4 ± 0.0 ^a^	12.7 ± 1.1 ^cd^	56.6 ± 1.1 ^g^	18.9 ± 1.8 ^e^
NE-Europe	4	< LQ	1.6 ± 0.0 ^b^	32.9 ± 0.0 ^f^	ND	11.4 ± 0.3 ^c^	6.0 ± 0.2 ^d^	0.6 ± 0.0 ^cd^	0.6 ± 0.9 ^a^	13.9 ± 0.3 ^d^	13.3 ± 0.8 ^cde^
NE-Europe	5	< LQ	3.5 ± 0.3 ^c^	3.7 ± 0.4 ^b^	0.8 ± 0.5 ^a^	2.9 ± 0.7 ^a^	3.4 ± 0.3 ^c^	0.4 ± 0.1 ^a^	4.1 ± 2.6 ^ab^	0.4 ± 0.2 ^a^	71.9 ± 2.7 ^f^
NE-Europe	6	< LQ	0.8 ± 0.0 ^a^	52.1 ± 1.2 ^g^	ND	7.8 ± 0.2 ^b^	6.3 ± 0.1 ^d^	0.6 ± 0.0 ^cd^	1.4 ± 0.2 ^a^	9.5 ± 0.8 ^c^	2.4 ± 0.1 ^a^
S America	7	0.3 ± 0.0 ^a^	0.8 ± 0.0 ^a^	ND	0.9 ± 0.0 ^a^	8.1 ± 0.1 ^b^	0.4 ± 0.0 ^a^	3.2 ± 0.1 ^g^	5.5 ± 0.1 ^ab^	1.3 ± 0.1 ^a^	18.0 ± 0.7 ^e^
S America	8	<LQ	<LQ	20.8 ± 0.1 ^d^	0.2 ± 0.0 ^a^	1.2 ± 0.0 ^a^	0.3 ± 0.0 ^a^	0.5 ± 0.0 ^b^	1.5 ± 0.1 ^a^	<LQ	9.0 ± 0.2 ^bcd^
SW-Europe	9	ND	ND	34.1 ± 0.9 ^f^	ND	2.8 ± 0.1 ^a^	0.8 ± 0.0 ^ab^	0.7 ± 0.0 ^e^	5.6 ± 0.3 ^ab^	33.3 ± 1.4 ^f^	7.6 ± 0.5 ^abc^
SW-Europe	10	ND	6.0 ± 0.1 ^e^	ND	ND	2.4 ± 0.0 ^a^	1.0 ± 0.0 ^ab^	0.3 ± 0.0 ^a^	6.3 ± 0.5 ^abc^	28.3 ± 0.2 ^e^	13.4 ± 1.0 ^cde^
SW-Europe	11	<LQ	6.5 ± 0.3 ^e^	6.3 ± 1.6 ^c^	7.3 ± 4.1 ^b^	12.2 ± 3.2 ^c^	2.9 ± 0.8 ^bc^	1.0 ± 0.1 ^f^	11.4 ± 0.4 ^bcd^	13.7 ± 0.6 ^d^	72.0 ± 3.1 ^f^
SW-Europe	12	ND	4.6 ± 0.4 ^d^	1.7 ± 0.4 ^ab^	0.4 ± 0.3 ^a^	2.0 ± 0.2 ^a^	1.9 ± 0.1 ^abc^	0.4 ± 0.1 ^a^	32.2 ± 7.4 ^e^	25.6 ± 2.6 ^e^	13.0 ± 2.7 ^cde^
SW-Europe	13	ND	6.6 ± 0.1 ^e^	ND	ND	3.0 ± 0.0 ^a^	2.3 ± 2.8 ^abc^	0.3 ± 0.0 ^a^	4.0 ± 0.2 ^ab^	33.9 ± 1.1 ^f^	110.6 ± 6.0 ^g^

^a–g^ different letters means significant difference (*p* < 0.05) for the same phenol compound; LQ: Limit quantification; ND: Not detected; * These compounds elute at the same retention time (RT), so they were quantified together. NE: North-East; S: South; SW: South-West.

## Supplementary Materials

The following are available online at https://www.mdpi.com/2076-3921/9/1/75/s1, Table S1: Limit of detection and quantification of phenolic compounds (mg/mL) by HPLC-UV, Table S2: Antioxidant activity against ABTS^•+^ radical (μmol Trolox/g) of standard phenolic compounds, Table S3: TEAC values (μmol Trolox/g) of the propolis samples calculated from the addition of the TEAC contribution of each phenolic compound quantified by HPLC-UV, and actual TEAC values of the propolis samples.
