# Supplementary material for: Phenolic Profile, Antioxidant Capacities and Enzymatic Inhibitory Activities of Propolis from Different Geographical Areas: Needs for Analytical Harmonization
Source: Antioxidants (Basel). 2020 Jan 15;9(1):75. doi: 10.3390/antiox9010075 (PMC7022753; doi:10.3390/antiox9010075)
Supplement: Supplementary file 1 [file antioxidants-09-00075-s001.pdf]

Table S1. Limit of detection and quantification of phenolic compounds (mg/mL) by HPLC-UV

Phenolic standard	LD	LQ
Gallic acid	0.0003	0.0009
Caffeic acid	0.0003	0.0011
Catechin	0.0018	0.0060
Clorogenic acid	0.0001	0.0043
p-Coumaric acid	0.0005	0.0018
Ferulic acid	0.0013	0.0044
Naringenin + Quercetin*	0.0007	0.0025
Apigenin + Kaempferol*	0.0017	0.0059
Pinocembrin	0.0013	0.0044
CAPE + Galangin*	0.0049	0.0163

Table S2. Antioxidant activity against $ABTS^{\bullet+}$ radical (μmol Trolox/g) of standard phenolic compounds

Samples	μmol T/g
Gallic acid	24173 ± 523
Caffeic acid	6503 ± 237
Catechin	5772 ± 129
Clorogenic acid	2380 ± 29
p-Coumaric acid	374 ± 7
Ferulic acid	7507 ± 66
Quercetin	5852 ± 89
Naringenin	20 ± 0.2
Kaempferol	4270 ± 12.9
Apigenin	43 ± 2.3
Pinocembrin	15 ± 0.4
Galangin	2697 ± 99
CAPE	3530 ± 231

Table S3. TEAC values (µmol Trolox/g) of the propolis samples calculated from the addition of the TEAC contribution of each phenolic compound quantified by HPLC-UV, and actual TEAC values of the propolis samples.

Propolis samples	Estimated range for TEAC according to phenolic composition by HPLC (µmol T/g)	Actual TEAC (µmol T/g)
1	305-339	398
2	214-289	453
3	113-185	468
4	286-303	427
5	266-345	426
6	363-374	363
7	70-126	466
8	147-164	280
9	225-259	465
10	84-124	467
11	317-431	466
12	93-242	466
13	360-471	470
